# Human Detection from a Mobile Robot Using Fusion of Laser and Vision Information

**DOI:** 10.3390/s130911603

**Published:** 2013-09-04

**Authors:** Efstathios P. Fotiadis, Mario Garzón, Antonio Barrientos

**Affiliations:** Centro de Automática y Robótica, UPM-CSIC. Calle José Gutiérrez Abascal, 2. Madrid 28006, Spain; E-Mails: ma.garzon@upm.es (M.G.); antonio.barrientos@upm.es (A.B.)

**Keywords:** human detection, unmanned ground vehicle, outdoors surveillance, sensor fusion, laser range finder, monocular vision

## Abstract

This paper presents a human detection system that can be employed on board a mobile platform for use in autonomous surveillance of large outdoor infrastructures. The prediction is based on the fusion of two detection modules, one for the laser and another for the vision data. In the laser module, a novel feature set that better encapsulates variations due to noise, distance and human pose is proposed. This enhances the generalization of the system, while at the same time, increasing the outdoor performance in comparison with current methods. The vision module uses the combination of the histogram of oriented gradients descriptor and the linear support vector machine classifier. Current approaches use a fixed-size projection to define regions of interest on the image data using the range information from the laser range finder. When applied to small size unmanned ground vehicles, these techniques suffer from misalignment, due to platform vibrations and terrain irregularities. This is effectively addressed in this work by using a novel adaptive projection technique, which is based on a probabilistic formulation of the classifier performance. Finally, a probability calibration step is introduced in order to optimally fuse the information from both modules. Experiments in real world environments demonstrate the robustness of the proposed method.

## 1. Introduction

The surveillance of critical infrastructure is heavily based on the detection and evaluation of human presence. Current systems usually employ a variety of strategically placed sensors and rely on human supervision. However, these systems suffer, due to the overwhelming amount of data, which are difficult to follow, and, moreover, from repetition that leads to mental and visual fatigue. In order to alleviate these problems, various methods for the automatic detection of suspicious actions have been proposed [1].

In large outdoor infrastructures, the magnitude of the area under inspection renders the task implausible to fulfill with static sensors alone. A distributed mobile robot system could help towards such a direction. Using autonomous navigation, robots can follow predetermined or random paths along the perimeter or in between the buildings of the infrastructure. In case of intrusion, detection algorithms can trigger an alarm or provoke further action. Moreover, the detection of the intruder allows his or her tracking and following, while keeping security personnel informed. In order to complete this task, the robotic platform needs to be equipped with an effective human detection module.

Detecting humans from a moving platform raises many difficulties. Outdoor environments are very noisy, and sensors can be affected by weather conditions, changes in illumination, movement or terrain irregularities, among many other factors. Constrains on the on board computational power together with the need of real time processing make the problem ever more demanding. Therefore, a robust and computationally-efficient set of algorithms is required.

This work presents a robust method for human detection on board an unmanned ground vehicle (UGV). The method is based on an information fusion scheme that uses a laser range finder and image data. Both sensors have their own advantages and drawbacks. Using them in a complementary way increases the overall performance compared to employing each one individually.

The laser data provide useful information about range and the geometrical characteristics of the surrounding objects. The segmentation of laser points into clusters and using a set of features to classify whether they belong to a human or not has been proposed [2]. Although this method has been proven efficient indoors, it is designed for human leg detection and uses distance-based features. An extension of this work is proposed here, intended to be used outdoors and with the premise of obtaining only one cluster per person. The distance-based features capture scene-specific information, and although very discriminative, they harm the generalization capability of the algorithm. In other words, the classifier learns the scene dependent features well, and it fails to deliver competent performance on different scenarios. Furthermore, the laser sensor is very prone to outdoor noise, and the features are dependent on the human pose. In order to better handle the information variations due to distance, human pose and sensor noise, a novel feature set has been proposed. It not only performs better outdoors, but at the same time, significantly improves the generalization of the system, making it capable of performing well in scenes that it was not trained to cope with.

Despite their usefulness, the information contained in the laser data is limited. Since the number of points returned is relatively low, the detection based solely on laser data may be unreliable. The camera vision signal is generally more informative and provides object texture and shape, which makes it ideal for human recognition [3]. Since no localization is provided, the whole image must be thoroughly processed, something that slows down the detection task significantly. The range information from the laser can be very helpful in localizing separate objects in the environment [4]. By exploiting the localization information of the laser, the computational time needed by the vision system can be reduced. Due to the geometrical setup, this localization can be well defined only on the horizontal axis. Surface abnormalities, steep inclinations, inherent vibrations or rapid acceleration of the UGV can introduce noise in the estimation. All these factors make the definition and projection of a correct region of interest (ROI) a non-trivial task [5]. In this work, this is addressed by a novel adaptive projection technique that uses *a priori* information about the classifier to construct a probabilistic detection model.

Fusion of the probabilistic outcome of the two sensor modules enables the extraction of non-redundant information. This is achieved by incorporating them in a probabilistic fusion framework that can be useful in difficult situations, such as occlusions, when one sensor alone cannot provide a correct detection [6]. In order to optimally combine the two outcomes and reduce the correlation between them, an off-line classifier calibration process is introduced.

The main contributions of the work presented here can be summarized by the following points:

- Laser detection: An extended feature set targeted for large outdoors environments, based solely on structural information, is proposed. The information variations due to distance, the human pose and sensor noise are encapsulated through feature normalization, providing better generalization.
- Vision detection: The problem of vertically localizing a projection from the laser to the image plane is addressed. A novel adaptive region of interest (ROI) projection technique helps compensate for misalignment, caused by otherwise uncontrolled factors, which often leads to false predictions.
- Information fusion: The redundancy and the correlation between the two classifiers is reduced by first calibrating them. The output probability of the fusion scheme is thus more optimal.

Our approach adapts and builds upon well established techniques in the human detection field. The techniques proposed have been assessed in detail, so as to provide a competent tradeoff between performance, scalability and execution speed time, when it is used on board a robotic mobile platform.

The paper is organized as follows. In the next section, previous work related to ours is briefly discussed. In Section 3, an overview of the proposed method is described. Detailed descriptions of the laser and vision detection modules are found in Sections 4 and 5, respectively. The information fusion methodologies are presented in Section 6. Then, Section 7 explains the experiments held in order to validate the method. Finally, Section 8 shows the results of the experiments, followed by our conclusions.

## 2. Related Work

During the few last years, applications of human detection have been proven attainable. Intelligent systems have improved the surveillance and safety of public places [1]. More recently, commercial cars have been equipped with active pedestrian detection and avoidance systems [7,8]. Similarly, such systems are being deployed in service robots [9].

Nevertheless, human detection is not an easy task. A lot of different methodologies using various sensor modalities have been proposed. The most commonly used sensor is a camera in a multitude of setups, such as monocular or stereo vision, sensitive to either the visible or the infrared light spectrum. The fact that computer vision can provide a rich three-dimensional signal, while remaining relatively inexpensive and easy to deploy, has led to the advancement of many different approaches in human detection in this field [3,10].

A plethora of these algorithms uses a *sliding window* approach. Dalal and Triggs [11] proposed a method for human detection that is based on this approach, with very good detection results at a comparatively efficient speed. The detection window is subdivided into cells, and for each one, a histogram of oriented gradients (HoG) is accordingly computed. The final detection is performed by a linear support vector machine (SVM) classifier. *Shape-based* methods use template matching algorithms to detect the appearance of human silhouettes on the image [12]. Another distinct approach is *part-based representations*. In this case, instead of exhaustively searching the image, this technique tries to learn and identify human parts and their relative positions [13]. A variation of the HoG descriptor is used in the vision module of this work (see Section 5.1).

Although significant advances have been introduced, vision-based systems suffer mainly from high computational requirements. Additionally, when real time applications are developed for systems like a small UGV, they are constrained to lower resolutions, due to the limited processing power and low payload capacity. As a consequence, the detection range is also limited. This work uses the laser range readings to reduce the search space and to comply with the real-time requirements of an autonomous surveillance UGV.

Laser range finders have been extensively used on autonomous ground vehicles for multiple tasks, such as object recognition and avoidance, or simultaneous localization and mapping (SLAM). The fact that human parts—especially legs—display distinct geometric characteristics in laser range data has been exploited for the detection and tracking of humans. These techniques generally rely on the segmentation of the two dimensional signal into separate *clusters* [14]. Consequently, for each cluster, various geometric and statistical characteristics are extracted. The identification can be made by either manually selecting fitting thresholds [15] or through machine learning [2]. Nevertheless, the information of the laser data is not enough to provide a set of highly discriminative features. Tracking of the moving object is commonly used in order to improve the performance. Human motion or gait models combined with tracking algorithms provide the necessary formulation for this task [4,16]. Another approach is based on occupancy grids, where the space is divided in cells, and the probability of each cell being occupied is computed based on information from current and previous laser scans [17]. Apparently, both tracking and occupancy grid methods are susceptible to occasions where humans remain stationary.

The work of Arras *et al.* [2] has been shown to be effective indoors. Using their method as a basis, this work proposes an extension aimed at being used in more diverse outdoor environments. The problem with feature-based methods arises from the use of characteristics dependent on the particular surroundings, such as the distance from the laser or the distance between objects. Although highly discriminative, these features tend to be scene-specific and to provide very poor generalization, a disadvantage that becomes more apparent in outdoor scenes that have greater variability. Furthermore, outdoors, the laser sensor is very susceptible to noise. Another finding is that the features are also dependent on the relative pose of the objects. In order to alleviate these phenomena, a novel feature set that captures the aforementioned dependencies is proposed in this work. The use of normalization techniques helps the classifier to adapt itself and discard intrinsic correlations, providing better overall outdoor performance. At the same time, it significantly increases the robustness of new scenes, which is critical in applications concerning large outdoor infrastructures.

Simultaneous use of both laser and computer vision has been used to provide a more reliable prediction, both in terms of detection accuracy and speed [18]. Applications of such bimodal detection systems have been introduced in mobile service robots [9] and automated braking systems for vehicles [19]. In various works, the range data from the laser are used in combination with geometric models of the surroundings to confine the search on the image data into areas of high interest [19,20]. The localization, provided by the laser data, assists in deducing ROIs that are subsequently processed and classified by the vision module. This approach helps to dramatically increase the execution speed, while at the same time, decreasing false alarms, because the detection is bounded to relevant areas.

However, the latter approach is limited in the sense that it does not take into account the laser discriminative information that could, in combination with the vision detection, provide more reliable results. With fusion techniques, information from both sources is evaluated; thus, partial incompleteness of the human appearance in one source can be addressed using the other. This is especially helpful in *hard* situations, when occlusions happen, due to the interaction between dynamic and static elements of the environment.

Fusion methods are generally divided into two categories, depending on which level the fusion is being done. In feature level fusion, the features for each laser cluster and vision region are computed separately and are then concatenated to a single vector used by a subsequent classification scheme [21,22]. In high or detector-level fusion, the data provided by each sensor lead to two separate classification results, and the combination of the results is done in a final fusion step. As before, laser data are used to reduce the vision module search space. Prior assumptions about human height and the geometric characteristics of the environment are used to define a projection ROI [6,23–26]. Other approaches are based solely on the geometric constraints of the system and the scene to construct a relevant space hypothesis [9,27,28].

Many of the methods that have been presented make use of fixed-size window projection. In this case, the vision module is highly susceptible to projection errors that occur when the platform is moving. Although the horizontal boundaries of the apparent cluster are easily defined by laser data, there are a lot of misplaced projections produced by factors, such as the acceleration of the platform, ground irregularities and steep slopes. In such cases, the assumption that the ground is level and the robot is neither rolling nor pitching does not stand true [25]. This becomes more important when a descriptor, like HoG, which is sensitive to the relative position of the human on the detection window, is used [23]. Furthermore, while these shortcomings are found on big mobile platforms, such as a car, a small-sized UGV is significantly more sensitive to them, due to its smaller form. In order to overcome this, ground plane extraction using a 3D laser scanner has been proposed with the objective of finding an accurate estimate of the platforms pitch and roll [23]. In this work, an adaptive projection scheme is proposed to address this problem; it is a simpler solution, which does not require the use of complex sensors or algorithms. The size of the projection window is increased to compensate for the misalignment, and the resulting ROI is thoroughly searched for human presence. The final prediction outcome is based on a probabilistic formulation that incorporates *a priori* knowledge about the classifier's performance. The result is a more versatile detection system that is capable of correctly detecting hard situations, even when the person is not on the same level with the UGV. At the same time, the computational cost is held low by restricting the search to a meaningful scale-space only.

Laser and camera fusion systems produce the final prediction probability using the score outcomes from the two modules. The accurate conversion of the classifier outcome to a probability distribution is therefore essential. Classifiers, like adaptive boosting (AdaBoost) and SVM, are commonly used in detection, but their outcome consists of uncalibrated scores [29]. When cascades are used, the sum of the weak classifier weights can be used to provide an estimate [9,23,25]. The logistic function has also been used to normalize the outcomes [26]. Here, this problem is addressed by calibrating the classifiers using a well-known method, thus generating more precise probability distributions [30].

## 3. Methodology Overview

This system is aimed to be used on board a UGV performing surveillance operations around a critical infrastructure. The main goal is to achieve the best detection performance possible while maintaining real time processing.

The UGV is equipped with a laser range finder and a camera sensor. In order to be able to use the range data of the laser together with the image from the camera, first, the sensors need to be calibrated, both intrinsically and extrinsically. This calibration process provides the translation and rotation vectors, which are needed for converting a point in the laser scan to a corresponding pixel in the image, as is described in Section 7.1.

**Figure 1.**
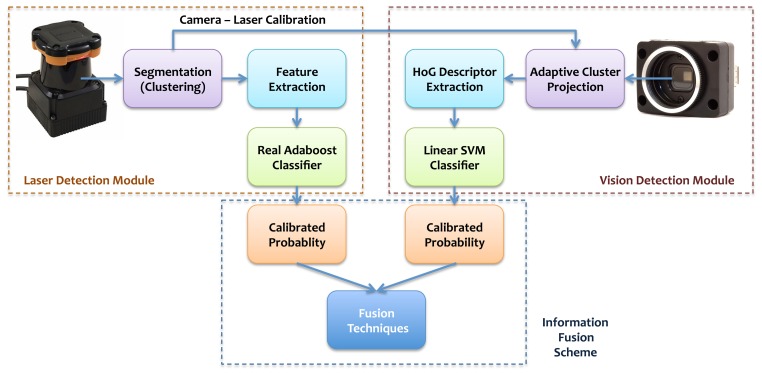
Schematic diagram of the proposed method.

The data readings from the two sensors are synchronized and then individually processed. Both modules, laser and vision, are comprised of two parts: a feature extraction algorithm and the subsequent pattern recognition process. Initially, the laser data are segmented into clusters, and a set of geometrical and statistical features is computed for each one. Afterwards, a Real AdaBoost classifier produces a probability estimate of whether it belongs to a person or not. Furthermore, these clusters are projected to the camera image for further processing in the vision module. For each projection, a corresponding ROI in the image is assessed using an adaptive window approach that utilizes a HoG descriptor and an SVM classifier. The probability outcome of both modules is subsequently merged into a single likelihood using fusion information techniques. Since the angular range of the laser range finder is much greater than that of the camera, some clusters do not have corresponding projections on the image. Nevertheless, those clusters are processed only from the laser module. A schematic overview of the method can be found in Figure 1.

## 4. Laser Detection Module

The laser detection module has a dual function. On the one hand, it provides the first estimation of human presence, which is later used in the fusion scheme. On the other hand, the range values from the sensor are used for localizing the apparent structures in the image plane, thus reducing the search space significantly

Data are acquired from the device as an ordered sequence of *n* points, *P* = {*p*_1_, *p*_2_, …, *p_n_*}. Each point is represented in Cartesian coordinates, *p_i_* = (*x_l_*, *z_l_*), derived from the polar coordinates, *ϕ*, *ρ*, of each range reading (*x_l_* = *ρ* sin *ϕ*, *z_l_* = *ρ* cos *ϕ*). In order to be consistent with the calibration, the laser readings are considered to belong in the plane where *y_l_* = 0.

### 4.1. Prepossessing and Segmentation

Individual points provide little to none structural information. Before any further processing is made, it is very crucial to obtain clusters representing distinct surrounding objects. Since the data points are sequential, a one-dimensional gradient filter mask [−, 1] can be applied to produce a vector of differences, Δ. Values greater than a given threshold, T_Δ_, define the borders of the clusters. This method is widely known as jumping distance segmentation. When big differences exist in readings between two consecutive laser points, it is very common to have a shadow effect, which means that the readings are erroneously somewhere between the two points. Methods based on Euclidean distance are greatly affected by this phenomenon. An appropriate adaptive filtering is applied beforehand in order to alleviate this effect. This is a relatively simple and fast algorithm; for an overview of segmentation methods and threshold estimation techniques, see [14].

**Figure 2.**
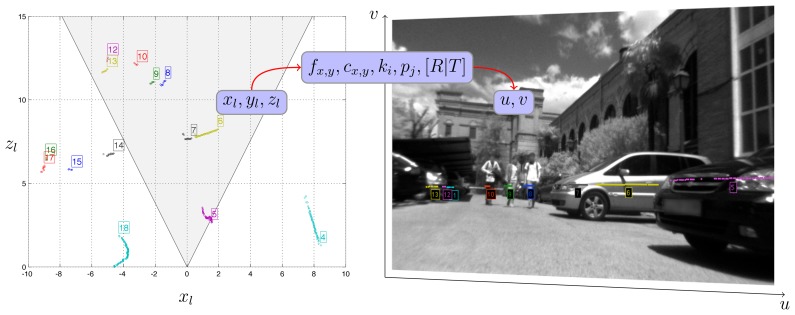
Projection of laser segmented clusters to the image plane using the calibration parameters. In the left image, the shaded area indicates the field of view of the camera. The color and number of each cluster are the same in both views.

This type of segmentation was originally conceived to work in indoor environments and normally leads to two distinct clusters, one for each leg. In our application, it is important to obtain a single cluster for each human in order to correctly project a region of interest in the image plane. This is partially achieved by placing the laser at upper leg height. However, as has already been discussed, this cannot be trusted, due to pitch variations of the UGV. Furthermore, outdoor conditions are much more versatile and affected by noise, which usually causes oversegmentation. Thus, a second cluster aggregation step is employed. When the euclidean distance between two distinct clusters is less than *T*_Δ_, they are united to form a new composite one. The value of *T*_Δ_ is the same as before. Very small remaining clusters that consist of less than three points are discarded as noise. In Figure 2, the segmentation of the laser data and their respective projection on the camera frame is shown.

### 4.2. Feature Extraction

After the segmentation has been completed, the next step is to extract a set of characteristics for each cluster. The feature extraction can be considered as a function where the input is the set of points of the cluster in *two-dimensional* Cartesian coordinates and the output is a vector in *n-dimensional* space, *f* : ℝ^2^ → ℝ*^n^*. In [2], Arras *et al.* propose a scheme for indoor human detection from laser data that is considered a work of reference in the field [6,23]. They use a combination of geometrical, statistical and distance-dependent features. Some of them are obtained by the cluster points themselves and others by their relationship with the surroundings, such as the distance from the sensor, the distance between clusters and the speed of the cluster. Those features do not conform to the aforementioned mathematical description, because the distance between objects cannot be inferred using the points of a sole cluster.

The aforementioned features have been built for describing human legs in indoor environments. The proposed extension is developed in order to produce a more appropriate feature set for the outdoor environment with the additional constraint of having only one cluster for each person. Since outdoors surroundings tend to be more complex, using features that are context specific, such as the distance between objects, harms the generalization of the detector. Furthermore, in outdoor areas, the distance between the laser and the targets can be large, so that far away objects may be represented by only a few points. The number of points of a human cluster is also dependent on other uncontrolled factors, such as the lighting conditions, the platform movement and the objects relative pose with respect to the laser. In order to study the variations on the feature values with respect to the aforementioned factors, normalization methods are employed. The distance from the camera and the number of laser points are used as normalization factors. The initial feature basis containing the distance-independent components that have been used in this work can be found in Table 1. From the initial feature basis, a final vector with 63 components is acquired as follows:

- The initial 13 features from the feature basis of Table 1.
- The initial 13 features divided by the distance from the origin.
- The initial 13 features multiplied by the distance from the origin.
- Features 2 to 13 divided by the number of points.
- Features 2 to 13 features multiplied by the number of points.

No distance-dependent feature is explicitly included in the final set. Using this novel extended feature set, the classifier employed in the subsequent step can learn and compensate for distance, noise and human pose-dependent variations, ultimately providing better generalization.

Three feature sets were considered for comparison reasons: the feature basis of Table 1, the feature basis with distance features added and the proposed 63-dimensional feature set. In Table 2a and 2b, a performance comparison between the three sets is presented, and the results are further discussed in Section 8.

### 4.3. Classification

The identification of the cluster is performed by an AdaBoost (*i.e.*, adaptive boosting) classifier. In boosting, a set of discriminative *weak* classifiers are combined to construct a final *strong* classifier [31]. Individual classifiers are called weak because the only requirement is to perform better than random guessing. Due to their simplicity, they are computationally inexpensive. Generally, AdaBoost techniques tend towards margin maximization between the classes. Overfitting of the data is very improbable in practice, and the classifier provides good generalization. There are various different schemes of the AdaBoost algorithm; an overview can be found in the work of Friedman *et al.* [32].

The training input consists of labeled data, (*s_i_*, *l_i_*), *i* = 1 … *N*, where *N* is the number of samples. Each sample consists of an *n*-dimensional descriptor, *s_i_* ∈ ℝ*^n^*, and an annotation label, *l_i_* ∈ {+1,−1}, denoting a positive or a negative example. In our case, the descriptor is a 63-dimensional feature vector, while the label signifies if the sample belongs to a human or not. In each iteration, the algorithm trains a weak classifier, which produces a weak hypothesis, *h_t_*(*s_i_*) : ℝ*^n^* → ℝ, over the weighted distribution, *D_t_*, of the training samples. Subsequently, the weight distribution is recalculated, taking into account the error of the previous step. With this method, the weight of the wrongly classified examples is increased, forcing the next classifier to focus more on those *hard* cases. The final strong classifier, *H*(*s_i_*), is produced by the weighted sum of the *T* best weak hypotheses. An outline of this procedure can be found in Algorithm 1.


**Algorithm 1** Outline of Real AdaBoost.
**Input:** Training data samples, (*s_i_*, *l_i_*), *i* = 1,2,…, *N*; *s_i_* ∈ ℝ*^n^*, *l_i_* ∈ {+1, −1}**Initialization:** Set uniform sample weights, 
$w_{1}(i)=\frac{1}{N}$.**Iteration:** For *t* = 1, 2, …, *T*, repeat the following steps:
Train the weak classifier to obtain a class probability estimate, *P*(*l* = 1∣*s*) ∈ [0, 1], using the weighted samples.Set $h_{t}(s)=\frac{1}{2}\log\frac{P(l=1|s)}{P(l=-1|s)}$.Update the sample weights: $w_{i}^{\left(t+1\right)}=w_{i}^{\left(t\right)}\exp(-y_{i}h_{t}(s_{i}))$, *i* = 1, 2, …, *N*.Normalize the weight distribution: $w_{i}^{\left(t+1\right)}=\frac{w_{i}^{\left(t+1\right)}}{\sum_{i=1}^{N}w_{i}^{\left(t+1\right)}}$.**Output:** The final strong classifier, 
$H(s)=\text{sign}\;\left(\sum_{t=1}^{T}h_{t}(s)\right)$


In their implementation, Arras *et al.* used a modified version of the Discrete AdaBoost algorithm with stumps as weak classifiers. This variation of AdaBoost uses a weak hypothesis with a response restrained to a discrete outcome, essentially two classes, *h_t_*(*s_i_*) : ℝ*^n^* → {−1, +1}. In our implementation, the Real Adaboost version has been used. It is reported to be less immune to noise and generalizes better in practice than its Discrete counterpart [31,32]. Furthermore, our data set is significantly larger than that of Arras *et al.*, and our experiments showed that using stumps results in a classifier that is too weak and that underfits the data. Therefore, our choice for a weak classifier is a two-split decision tree. Finally, our proposed feature set yields better results when more classifiers are used, so empirically, *T* was set to 20. These changes provide considerable performance improvements and better generalization at a very low speed cost (see Section 8).

The low cost comes also from another advantage of AdaBoost classifiers. After the training procedure has been completed, the classification is based on a smaller subset of the complete feature vector. Since this knowledge is *a priori* available, the feature extraction algorithm can be accordingly adjusted to only compute the appropriate features, thus leading to faster execution time.

## 5. Vision Detection Module

The vision detection module serves as a second opinion observer that helps to improve the overall detection performance of the system. The detection is based on the established method of the histogram of oriented gradients. A novel adaptive projection method has been developed in order to obtain an accurate ROI from the image.

### 5.1. Histogram of Oriented Gradients

Edge orientation histograms have been extensively used in the computer vision field for object detection. Lowe's *Scale Invariant Feature Transformation (SIFT)* combines gradient orientation information with local spatial histogramming in a sparse grid [33]. The *histogram of oriented gradients* descriptor, proposed by Dalal and Triggs, uses a dense overlapping grid along with local contrast normalization and has been proven very effective for human detection in images [11].

**Figure 3.**
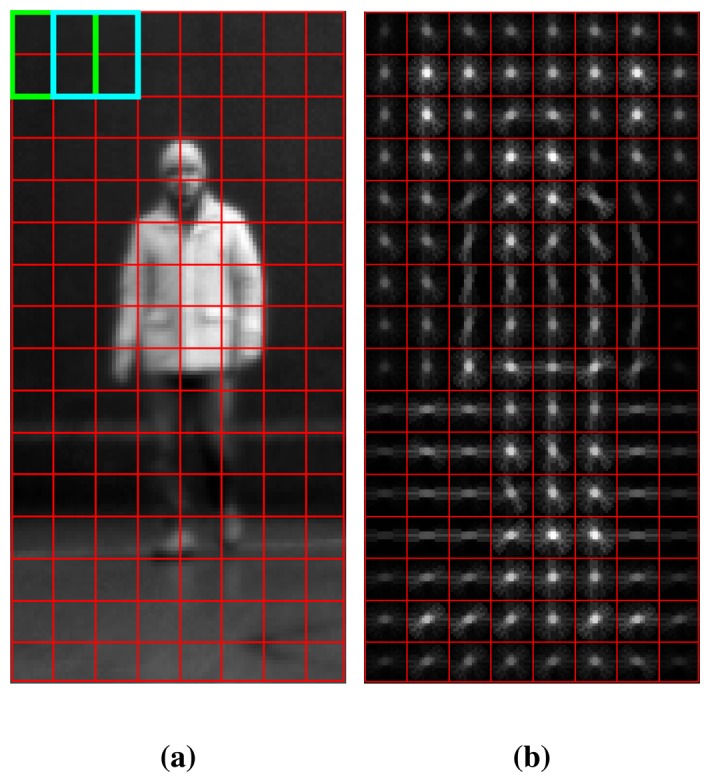
Histogram of oriented gradients descriptor **(a)** The histogram of oriented gradients (HoG) descriptor is computed over image cells (in red) and 2 × 2 overlapping blocks of cells (green and cyan); **(b)** visualization of the HoG descriptor computed for the same image

Though HoG entails a lot of details and parameters, a brief description of the algorithm is provided here. The parameters used in our work have been reported to provide the best results and are considered as *standard* in recent implementations. The descriptor is evaluated over a fixed-size sliding window of 128 × 64 pixels. First, the magnitude and orientation for each pixel of the image is computed. The window is divided into smaller regions called *cells.* For each cell, a nine-bin local histogram over the gradient orientations is composed with the magnitude of the gradients serving as a weighted vote. Contrast normalization of local responses is applied over larger spatial regions in order to overcome illumination variations, called *blocks.* The block dimensions are 2×2 cells, and the grid is overlapping, so each cell finally contributes in more than one block. Before being fed to the SVM classifier, the features are concatenated into a 3,780 dimension vector. The partition of a region into blocks and cells and the corresponding oriented histograms can been seen in Figure 3.

### 5.2. SVM Classifier

The evaluation of the HoG descriptors is performed by a linear support vector machine classifier. SVM is a machine learning algorithm, proposed by Cortes and Vapnik [34], that has been extensively used in pattern recognition applications. The linear SVM method conceptually follows and extends the idea that input vectors, *s_i_* ∈ ℝ*^n^*, can be effectively separated by a hyper plane, *w^T^w* + *b* > 0, lying on the same *n*-dimensional space. It mainly focuses on finding the hyper plane that achieves the maximum separation between the two classes. For that, it is also known as a maximal margin classifier. By using kernels, such as polynomials or Radial Basis Functions, the data can be mapped into higher dimensions, *f* (*s_i_*) : ℝ*^n^* → ℝ*^m^*, *m* > *n*. This allows further insight into higher order feature spaces. Finally, since the method is less susceptible to outliers, it has been proven to be very effective on non-separable classes.

### 5.3. Adaptive ROI Projection

The laser module performs the segmentation of the laser data into separate clusters, described in Section 4.1. Each individual cluster is, afterwards, projected into the image plane using the parameters obtained by the laser—camera extrinsic calibration, as explained in Section 7.1 and shown in Figure 2. Considering that the field of view of the camera is much smaller than the angular range of the laser range finder, it is apparent that only a portion of the clusters can be accordingly projected.

From the optical theory, the distance of an object with known dimensions can be calculated from its projection on the sensor. If the procedure is inverted, it is possible to obtain the height in pixels of an object, when its distance and dimensions are known, as described by Equation (1).

(1) $$\text{ROI Height}(\text{pixel})=\frac{\text{Object Height}(m)\times \text{Frame Height}(\text{pixel})\times \text{Focal Length}(m)}{\text{Distance}(m)\times \text{Sensor Height}(m)}$$

In Equation (1), ROI height is the wanted height of the projection window in pixels, object height is the corresponding height in the physical world, the frame height is the sensor's pixel resolution in the corresponding axis, focal length is the intrinsic lens parameter, distance is the distance of the cluster's centroid from the camera and sensor height is the camera's physical sensor dimension. The range information from the laser and the extrinsic calibration provide the distance of the object's centroid from the camera. An approximation of the object dimensions can be acquired by taking into account the medium human height and also by using some detailed information on how the HoG descriptor classifier is trained.

**Figure 4.**
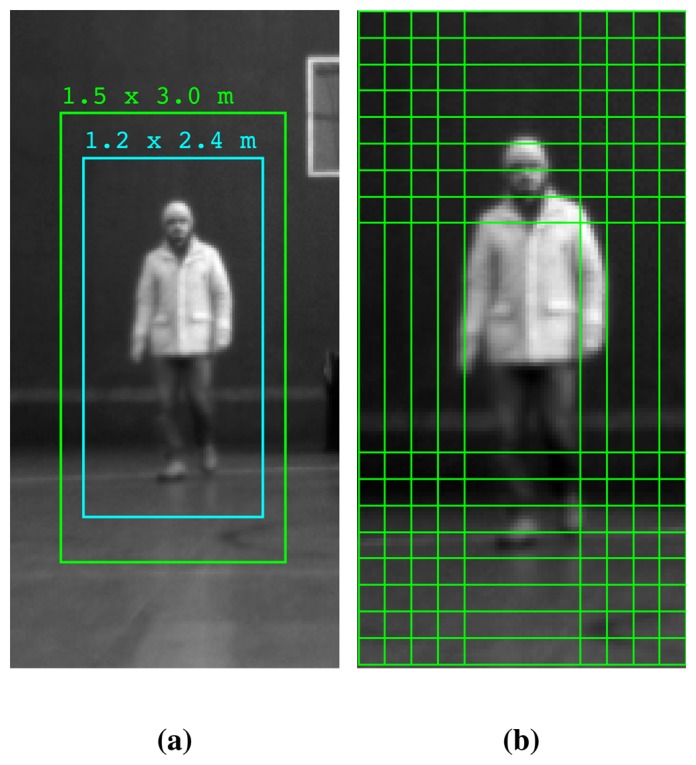
Fixed and adaptive projection methods **(a)** Projection of the same cluster for different corresponding sizes. The cyan rectangle is used in the fixed case, and the larger green window is used in the proposed adaptive method. **(b)** In the adaptive method, the region of interest (ROI) is scanned by an overlapping sliding window over multiple scales. In this figure, the maximum scale of the pyramid is depicted and the sliding window is of the size 2.0 × 1.0 meters.

The dimensions of the training images are 128 × 64 pixels, and according to the creators, there is a sixteen pixel margin around the person [11]. This means that the human figure occupies 75% of the image height. In Section 7.3, INRIAand MIThuman databases are described in more detail. According to recent statistics, medium human height is estimated to be 175 ± 15 *cm* [35]. The projection window dimensions can be estimated by adding the margin used for the training data set and taking into account the variations around mediums. These estimations range from 2.0 × 1.0 *m* to 3.0 × 1.5 *m.* It is worth noting that, since HoG is computed over a region of constant dimensions, the maximum effective distance is linearly related to the projected window size. This can be illustratively seen in Figure 4a.

It is evident that strictly defining the ROI size leads to some misclassifications, due to human height variations. Furthermore, the laser data can only confine the extremes of each object horizontally. The vertical boundaries can be estimated by taking into account the distance of the object and the height of the laser sensor with respect to the ground. Unfortunately, this hypothesis only stands true for even surfaces and while the UGV is static. When the platform is moving, pitch changes are very probable to occur, due to inherent vibrations, acceleration or deceleration. Furthermore, terrain irregularities, steep inclination and other uncontrolled factors may produce the same effect.

A novel method of adaptive projection has been used in order to restrict these effects. Instead of using a fixed-size window, a pyramid of meaningful sizes is constructed and thoroughly searched for possible human presence. Additionally, the use of an enlarged projection window increases the operational range of the vision module, thus producing overall more reliable results.

Initially, a 3.0 × 1.5 *m* ROI is extracted from the image, according to the distance of the cluster. Next, the HoG descriptors are computed with a sliding window of dimensions 128 × 64 over an overlapping grid with a step of eight pixels in each direction. A visualization of the overlapping sliding window can be seen in Figure 4b. As previously mentioned, there is no use in probing regions of size less than 2.0 × 1.0 *m.* Therefore, in this first step, if the sliding window corresponds to a size smaller than this threshold, the original region is scaled down in pixels accordingly. This significantly speeds up the process by eliminating searches in a space of irrelevant size. Afterwards, the extracted region is scaled down by a predetermined factor, and the procedure continues iteratively until the sliding window reaches the final scale of 3.0 × 1.5 *m.* Finally, the pyramid of the descriptors is evaluated by the previously trained SVM classifier.

The evaluation over a large number of sliding windows, typically contained in a region, will likely contain some false predictions. Methods, like non-maximum suppression and mean shift, have been proposed in order to cluster multiple detections when no prior knowledge is available [36]. In order to take into account the extra information available from the range data, a new way to discriminate between positive and negative examples is proposed. As previously mentioned, the classifier used has a high rejection rate, which produces only a few false alarms. Therefore the space-scale pyramidal search can be approached from a statistical perspective as a *Bernoulli process* with a product space given by *P* = {*p*, 1 − *p*}^ℕ^, where *p* is the possibility of success and *N*, the number of trials. Consequently, the evaluation of each sliding window corresponds to a *Bernoulli trial.* The probability of *success rate* can be approximated by the true negative rate of the SVM classifier on a representative test set. From the above results, the probability of having *k* true negative predictions out of *n* trials is given by the following equation:

(2) $$P(X=k)=\left(\begin{array}{c} n\\ k \end{array}\right)p^{k}{\left(1-p\right)}^{n-k}$$

In Equation (2), *n* is known, so it is possible to deduct the number of trials, *k_thr_*, so that *P*(*X* > *k_thr_*) ≤ 1 − *T_P_*. In practice, the value of the probability threshold, *T_P_*, is not very important, provided it is sufficiently small to reject random false alarms. If the number of trials classified as belonging to the positive class is greater than *k_thr_*, then the output of the algorithm is positive.

Since a multitude of sub-windows is evaluated for each cluster, there is an increase in the computational cost of the algorithm. Nevertheless, this increase is not significant enough to create a bottleneck and, thus, does not limit the real time functionality of the method. An outline of the method is shown in Algorithm 2.


**Algorithm 2** Outline of adaptive projection window method.
**Input:** A 3.0 *by* 1.5 m image region *I* ∈ ℝ^2^*^N^*^×^*^N^* and *T_P_* ≪ 1 probability threshold, where *N* is the width of *I* in pixels.**Initialization:** If the filter sliding window, *S*, of 128 × 64 pixels corresponds to less than 2.0 *by* 1.0 m, then, accordingly, scale down *I.***Procedure:** **while**
*S* corresponding dimension ≤ 3.0 *by* 1.5m **do**  *n* = 0;▹ number of total pyramid windows  **for**
*i* = 1; until *S* traverses all *I* vertically; *i* ← *i* + 8 **do**   **for**
*j* = 1; until *S* traverses all *I* horizontally; *j* ← *j* + 8 **do**    *H_n_* ← HoG descriptor of *S*(*I_ij_*)    *C_n_*, *P_n_* ← class and probability output of linear SVM classifier for input *H_n_*    *n* ← *n* + 1   **end for**  **end for**  Scale down *I* by a constant scaling factor, *F*. **end while** Find *k* for which 
$P(X>k)=\left(\begin{array}{c} n\\ k \end{array}\right)p^{k}{\left(1-p\right)}^{n-k}\leq 1-T_{P}$ **if**
*sum*(*C_n_* == +1) > *k*
**then**  *C_out_* ← +1  *P_out_* ← *max*(*P_n_*) **else**  *C_out_* ← −1  *P_out_* ← *min*(*P_n_*) **end if****Output:** The output class, *C_out_*, and probability, *P_out_*


## 6. Information Fusion Scheme

In the fusion module, the output prediction is improved by combining information from the two different sources in a meaningful way. The process of correlating data between the two sensors is addressed with the adaptive ROI projection described in Section 5.3. This preliminary fusion step ensures the correspondence between the outcome of the sensors, permitting further exploitation of their relationship.

The decentralized fusion scheme employed in this study uses probability estimations derived from each detection module. Initially, the outputs of each individual detector have to be mapped to a calibrated probability distribution. After that, three different approaches of probabilistic fusion are assessed to obtain a final estimation of certainty.

### 6.1. Calibrated Probabilities

The fusion scheme is focused on providing increased accuracy by integrating the two distinct detection methodologies described previously. Since the techniques are based on probabilistic estimates, the accuracy of those estimates is very important.

Classifiers, like SVM and AdaBoost, tend to maximize the margin between the two classes, and their calculated scores are shifted away from the typical range of probability values. This bias creates a distorted probability distribution. A method, *f*, for transforming the classifier score, *x*, to posterior probability is necessary in order to overcome this inconsistency:

$$x\in (-\infty ,\infty )\overset{f}{\to }P(y=1|x)\in (0,1)$$

Considering support vector machines, Platt [29] proposed that this could be accomplished by passing the output of the classifier through a parametric sigmoid function:

(3) $$P(y=1|x)=\frac{1}{1+\exp(Ax+b)}$$

The parameters of the sigmoid are fitted post-training, using maximum likelihood estimation on a calibration set (*x_i_*, *y_i_*), as shown in Equation (4). Gradient descent is used for the minimization process.

(4) $$\begin{array}{l} \;\underset{A,b}{\arg\min}\left\{-\sum_{i}y_{i}\text{log}(p_{i})+(1-y_{i})\text{log}(1-p_{i})\right\}\\ \text{where}:\\ \;p_{i}=\frac{1}{1+\exp\;(Ax_{i}+b)} \end{array}$$

The statistical interpretation of AdaBoost dictates that calibrated probabilities can be obtained by logistic correction. In this case, the theoretically proven parameters of the sigmoid are *A* = –2 and *b* = 0 [32]. This method works in the case of decision stumps, but when decision trees with more splits are used, the calibration tends to be poor. Similarly to SVM, Platt's scaling method has been experimentally tested with AdaBoost and other large margin classifiers, providing good calibration results [30].

### 6.2. Fusion Techniques

A probabilistic framework is incorporated to combine the outputs of the classifiers. The outputs inferred from the sensor data, *z*, are considered as conditional probabilities that depend on the underlying state, x, of the object under examination. Their likelihood is denoted as Λ*_i_*(*x_j_*) = *P*(*z_i_*∣*x_j_*), where *i* is the label of the sensor, *z_i_* ∈ {*z_l_* ≡ laser, *z_c_* ≡ camera}, and *j* corresponds to the states, *x_j_* ∈ {*x_1_* ≡ person, *x*_2_ ≡ not person}. When an observation is made, the sensor reading is considered fixed, and *i* is known; thus, the likelihood function, Λ*_i_*(*x_j_*), is considered a distribution in *x_j_*.

A reasonable hypothesis is to assume the same confidence for both sensors, so the same weight is assigned to them in every fusion technique. Their likelihood is finally combined to compute the posterior probability, which corresponds to the presence of a person, *P*(*x*_1_∣*z_l_* ∩ *z_c_*). Three different formulas—maximum, average and Bayesian-average—are evaluated for the purposes of comparison. The first two are simple and straightforward. The maximum formula is expressed as:

(5) $$\text{Maximum}(P(x_{1}|z_{l}\cap z_{c}))=\underset{i=l,c}{\max}(\Lambda_{i}(x_{1}))$$

while the average formula is:

(6) $$\text{Average}(P(x_{1}|z_{l}\cap z_{c}))=\frac{\sum_{i=l,c}\left(\Lambda_{i}(x_{1})\right)}{2}$$

The third fusion technique is inspired and derived from the Bayes theorem. In this formulation, a posterior distribution, *P*(*x*_1_∣*z_l_* ∩ *z_c_*), can be expressed as the normalized product of the likelihoods, Λ_i_(*x*_1_), given the presence of a person. From Bayes theorem, this can be directly written as follows:

(7) $$P(x_{1}|z_{l}\cap z_{c})=\frac{P(z_{l}\cap z_{c}|x_{1})P(x_{1})}{P(z_{l}\cap z_{c})}$$

The joint distribution, *P*(*z_l_* ∩ *z_c_*∣*x*_1_), must be known for computing the posterior probability, which is generally a difficult and complex task. In practice, the information obtained by different sources, *z_l_* and *z_c_*, can be considered *independent* when conditioned on the real underlying state of the object. Although this condition does not always stand true, it facilitates the computation of the combined posterior probability for different values of the state. The resulting equation is known as the *independent likelihood pool* [37]. Moreover, Equation (7) depends on prior information, *P*(x_j_), representing any previous knowledge about the occurrence of the states. Under the strong assumption that individual states are described by a *uniform prior*, 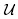
(*x_j_*), the final formula is devised:

(8) $$\text{Bayes}(P(x_{1}|z_{l}\cap z_{c}))=\frac{\prod_{i=l,c}\Lambda_{i}(x_{1})}{P(z_{l}\cap z_{c})}$$

where the denominator constitutes the marginal distribution, which acts as a normalizing factor, and can be defined as:

(9) $$P(z_{l}\cap z_{c})=\sum_{j=1}^{2}\prod_{i=l,c}\Lambda_{i}(x_{j})$$

## 7. Experiments

In this section, the platform used for testing our methodology is briefly described. First, the laser-camera calibration procedure is described. Then, the experiments carried out to collect the data sets are explained in detail. Furthermore, it also describes how the classifier training was made and which evaluation criteria were used.

### 7.1. Calibration

A correct calibration of the sensors is very important for the fusion implementation, because it serves two purposes. First, the intrinsic calibration provides the focal length (*f_x_*, *f_y_*) and principal point (*c_x_*, *c_y_*) of the camera sensors. It also provides the necessary radial and tangential parameters to compensate for inherent lens distortion (*k*_1_, *k*_2_, *k*_3_, *p*_1_, *p*_2_). Additionally, extrinsic laser-camera calibration is used to obtain the requisite rotation and translation matrices (*R*, *T*), which allow the transformation of a point belonging to the laser plane, into the image sensor pixel coordinates.

**Figure 5.**
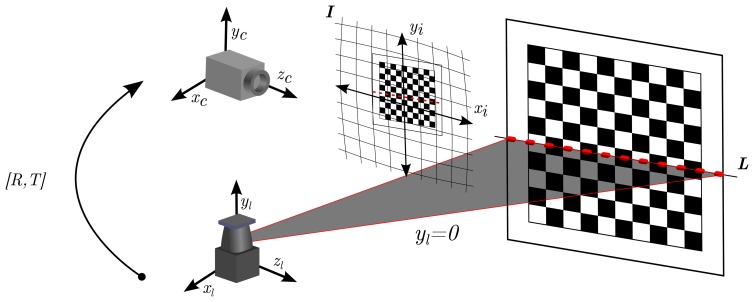
Calibration of the laser and camera Two reference frames are shown, one with respect to the camera (*x_c_*, *y_c_*, *x_c_*) and another with respect to the laser (*x_l_*, *y_l_*, *x_l_*). The extrinsic calibration provides the matrices, *R*, *T*, containing the rotation and translation between the two frames. *I* corresponds to the 2D image plane. The laser plane, *L*, is defined by *y_l_* = 0.

The procedure is formulated by the pinhole camera model, which has been implemented with a numerical precision of up to sixth order coefficients. First, using the translation and rotation matrices, the points are converted from the laser frame to a frame fixed with respect to the camera. Next, the points from the camera frame are perspectively projected to the image plane. The projection to the image plane is distorted, due to imperfections of the lens. This can be compensated for by undistorting the image using the distortion coefficients provided by the intrinsic calibration. Finally, the undistorted image plane coordinates are transformed into the equivalent pixel coordinates of the sensor using the intrinsic camera parameters of focal length principal point. The procedure is depicted in Figure 5.

A reference object, such as a flat checkerboard, is used for the calibration process. In order to record the calibration data, the object is positioned at various distances and orientations with respect to the sensors. Consequently, the normal vector of the checkerboard is extracted for every image, and the matching line parameters are computed from the range data. Therefore, to minimize the discrepancy between them, Zhang and Pless [38] proposed a two-stage process. First, a linear estimation is computed in order to get a rough estimate of the *R* and *T* matrices. Then, their final values are obtained by the optimization of a quadratic error function. Further improvements in the automatic object extraction and optimization can be found in [39].

### 7.2. Platform

**Figure 6.**
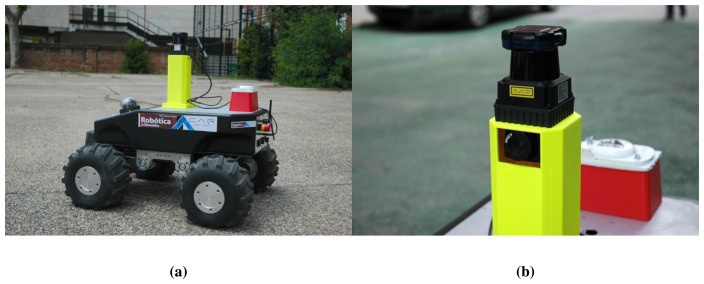
Platform and sensors used in experiments. (**a**) Summit XL mobile platform; (**b**) Hokuyo laser range finder above the Firefly camera.

The proposed methodology was assessed using data acquired on board a *Summit XL*™ mobile robotic platform developed by *Robotnik™*. It is equipped with a range of active and passive sensors, such as an IMU, a differential GPS, a pan-tilt-zoom camera and a laser range finder, among others. The maximum speed of the platform, which can be seen in Figure 6a, is three meters per second.

In our setup, a *Hokuyo UTM-30LX-EW*™ is used. It is a laser range finder that provides a measurement range of 30 meters and an angular range of 270° degrees with 40 Hz (scans per second) acquisition frequency. All data points have a fixed angular resolution of 0.25° degrees. The data are transferred through the TCP/IPprotocol and are collected using a driver built upon the communication specification protocol of the laser (SCIP). Data points are represented by polar coordinates (*ϕ*, *ρ*).

The vision system consists of a monochrome *Firefly MV*^®^ camera by *Point Grey*™ with a resolution of 752 × 480 pixels, a horizontal angle of view of 59° and a frame rate of 60 FPS. The camera uses the IEEE1394 (FireWire) protocol to send the data through a USB cable. The acquisition was made with a driver developed using a Linux APIfor FireWire cameras. Automatic CMOSsensor parameters were used where possible.

Before any data acquisition, the sensors were calibrated both intrinsically (camera) and extrinsically (camera-laser) with the procedure described in Section 7.1. Both sensors stamp their data with the acquisition time to enable the synchronization between scans and frames. Figure 6b shows a detailed image of the two sensors and their geometrical configuration.

### 7.3. Data Acquisition

The data were gathered while manually driving the platform inside the campus of the School of Industrial Engineering of the Polytechnic University of Madrid (ETSII-UPM). Three different datasets were collected in two different places. The first two sets of data were collected in the campus gymnasium, the first while the platform was held static and the second while it was moving. Although being a closed environment, the gymnasium was illuminated with dispersed natural light under cloudy conditions. In order to record the third set of data, another experiment was carried out in an outdoor area of the campus, where, usually, a lot of pedestrians pass by. Throughout this experiment, the platform was moving. Lighting conditions consisted mostly of direct sunlight with temporary cloud interference.

Inside the gym, in the static scenarios, only one person, following a predetermined route, passed in front of the robot each time. In the moving scenarios, a random number of people passed in front of the robot while it was moving. The people were walking in random paths, and in many cases, occlusions have happened, when people have crossed with each other. In one specific scenario, the people were standing still, while the robot was moving. There were no obstacles between the robot and the people walking in the gymnasium experiments. Outdoors, the experiments were held in a university campus area, and the people were walking in front of the moving robot in various directions. Some people were static and others at a level different from the robot. Furthermore, more occlusions happened when people crossed with each other or when they interacted with other surrounding elements. The robot's speed in the gymnasium was 0.25–0.50 m/s and outdoors, 0.50–0.75 m/s.

The complete set of data consists of 21,550 time-synchronized pairs of laser scans and image frames. The laser scans were segmented into clusters using the algorithm described in Section 4.1. The segmentation threshold was initially estimated using methods from [14]. These methods are more suitable for discerning between human legs. Since the creation of one cluster per person is important for the subsequent projection, the threshold was experimentally adjusted to the final value of *30 cm*. After the segmentation, a total of 485,420 laser clusters were produced, 43,040 of which belong to humans. Annotation of the clusters was done manually, aided by vision images where possible. To accelerate the annotation process, an assistive algorithm was developed. Our entire set corresponds to more than 625 seconds of real world data recordings. Altogether 28 distinct people appear in all three scenarios. A detailed description of the data sets can be found in Table 3.

As has been noted, the vision module only takes part in the classification of a portion of the segments produced by the laser. The number and the quality characteristics of these clusters vary, depending on which projection size and method are used. In this work, two different methods are compared. The first one is the fixed projection window, and the other one is the proposed adaptive projection algorithm. Our experiments have shown that in the fixed-width case, a size of 2.4 × 1.2 m gives the best results. In the adaptive projection case, the size of 3.0 × 1.5 *m* corresponds to the maximum scale of the pyramid.

From Equation (1), the range of the fixed window is calculated between 3.33–12.5 *m*, while that of the adaptive projection is 4.17–15.63 *m*. This increased range means that the critically more distant clusters are evaluated by both detection modules, something that increases the overall detection performance and the robustness of the method. Apart from the farther reach, the projective window approach also increases the operational range from 9.17 *m* to 11.46 *m*. This is the reason why more clusters are evaluated overall by *both* modules. Since the fusion of the two outcomes generates better results, this can be considered another advantage over the fixed-size projection. The difference in the evaluated clusters is depicted in Table 4, where the fusion data sets corresponding to the adaptive projection method are significantly larger that their fixed method counterparts.

### 7.4. Classifier Training and Calibration

Two distinct classifiers were trained and calibrated, one for each detection module. For the laser module, a Real AdaBoost classifier is used. It is made up of twenty weak classifiers, namely two-split decision trees. This setting is chosen, after several experiments, on the grounds of performance *versus* computational cost tradeoff, while trying to avoid overfitting, as well. Three different feature sets are compared. The first one is composed of the feature basis of Table 1. The second one is derived by the addition of distance-based features to the feature basis, and the third consists of the proposed 63-feature vector that is described in Section 4.2.

The stratified two-fold cross validation (holdout method) is used to produce the results. The training and test sets are derived from the complete data sets of Table 3. First, the training is done on the training set and the evaluation on the test set, followed by the reverse order. This way, each cluster on the test set is used both for training and evaluation. A separate classifier is trained and tested for each one of the three data sets.

Furthermore, the generalization properties of the method are also tested. A classifier is trained using the complete data set from the gymnasium moving scenario. It is then tested on the data from the outdoors scenario. The procedure is also done *vice versa*. This gives an insight of how well each feature set can adapt to a new environment.

For the vision module, two established, openly available databases were used. The first one is the MIT pedestrian image database, which consists of 924 pedestrian images (positive) limited to frontal and rear views [40]. The second one is the more challenging and complete INRIA person database [11]. The picture quality of the databases is very similar to the one produced by the camera used in our experiments. The rationale on using established databases over a proprietary data-set is because they cover a large number of human poses in a large variety of scenes that would be very difficult to replicate within our experiments. Using a restricted database could possible harm the generalization of the method, and since the objective of the proposed system is to be applied in a diverse environment, a more versatile approach was adopted.

The INRIA training set contains 2,416 positive images and 1,218 negative images, and the test set contains 1,126 positive and 453 negative images. Positive images from both databases are scaled to a size of 128 × 64 pixels, with the person located at the center of the image. Around the person, there is a sixteen pixel-wide margin. People appear in various poses and orientations in a broad range of backgrounds. From each non-pedestrian image of the INRIA database, a number of negative cropped images are randomly extracted, both for the training and the testing sets.

The HoG descriptors, computed for each image of the dataset, were used to train an SVM classifier. After some initial tests, it was observed that non-linear kernels show very small improvement—less than two percent—in comparison with the linear case. This improvement was accompanied by a disproportional increase in the computational cost. In their work, Dalal *et al.* [11] have drawn analogous conclusions. Furthermore, our results have not shown observable overfitting, neither in the initial cross validations tests nor with the final test set. Under the assumption that the database is uncorrelated, this can be attributed to the relatively high ratio between the input vector dimension and the training dataset size. For such reasons, the implementation used in this work is based on the linear version of the method. The only choice that had been made was the value of *C*, which modulates the importance of outliers lying beyond the support vectors. Using a relatively small value (0.01) means putting less importance on outliers and results in the training of what is commonly mentioned as a *soft* SVM classifier.

The training procedure consists of two steps. First, a preliminary linear SVM classifier is individually trained using the randomly extracted negative samples described above. Afterwards, all the negative images are exhaustively searched over the space-scale pyramid using the preliminary classifier. Every example that is falsely classified as one containing a human (false positive) is considered a *hard example*, and it is added to the negative training pool. The final SVM classifier is trained using the extended negative training set constructed in the previous step. These preliminary and final training sets along with the test set can be seen in Table 5. The final classifier is evaluated on the aforementioned test set, and the resulting confusion matrix is presented in Table 6. Note that the true negative ratio in this table serves as an approximation of the probability, *p*, in the binomial distribution expressed in Equation (2).

After the training of the classifiers, the probability calibration is necessary in order to obtain accurate fusion results. As explained in Section 6.1, Platt's scaling method is used for extracting the parameters, *A* and *b*, of the sigmoid in Equation (3). As a calibrating set, the test sets of each classifier are used. The results for the SVM are *A* = −3.82, *b* = 0.56 and for the AdaBoost, *A* = −1.78, *b* = −0.067, accordingly. It is worth noting that when stumps (one-split decision trees) were used in our experiments as weak classifiers for boosting, the theoretically expected values of *A* = −2, *b* = 0 were experimentally verified.

## 8. Results

The results of a classifier are often presented in the form of confusion matrices. Unfortunately, when several data sets are used, confusion matrices become relatively difficult to compare. In order to facilitate the comparison of results, a combination of three well-known metrics that capture the classifier behavior in total are used: precision, recall and specificity. Recall is otherwise referred to as sensitivity or the true positive rate, while specificity is also known as the true negative rate.

For the visualization of the results, various modalities exist in the literature, such as the receiver operating characteristic (ROC) curves and precision-recall graphs. In our work, detection error tradeoff (DET) graphs are chosen in order to simplify performance comparison. DET plots are variants of ROC curves that provide better insight when a particular operating range is of principal interest [41]. The false positive rate between 10^−3^ and 10^−1^, which is the most important in our case, is well articulated using this method. It thereby facilitates comparison.

### 8.1. Laser Module Results

First, the laser module on the complete acquired data sets, as described in Table 3, is evaluated. Three feature sets are used to train two distinct Real AdaBoost classifiers in each scenario: the basis feature set of Table 1, the basis with distance features added (Basis + DIST) and the proposed feature set.

It is observed that, regardless of the feature set used, the experiments carried out indoors give better results than those performed outdoors. Likewise, static experiments in the gym are *easier* than the moving experiments in the same scene. This can be attributed to the different complexities of the scenario and to the fact that outdoors, there is more noise in the data, due to direct sunlight illumination, terrain irregularities and other factors.

**Figure 7.**
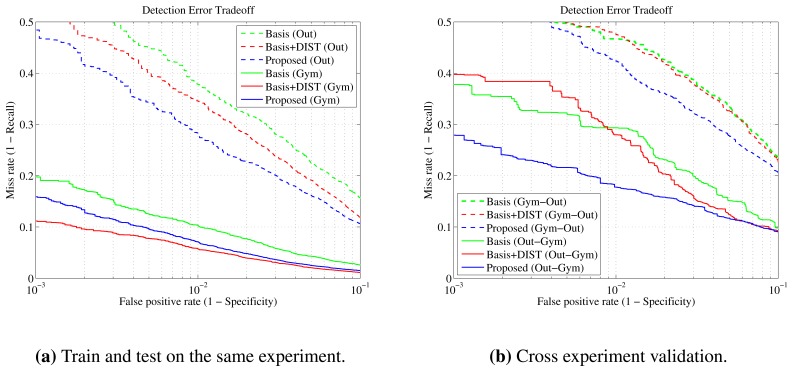
Laser feature sets—detection error tradeoff curves In both figures, the green line corresponds to the basis feature set, the red line to the basis plus the distance features and the blue line to the proposed feature set. (**a**) The training and test sets are acquired from the split up of same experimental data: solid lines indicate the gymnasium and dotted lines the outdoors data sets, respectively. (**b**) Solid lines signify that the outdoor data set is used for training and its gymnasium counterpart for testing. Dotted lines signify the reverse procedure.

More specifically, the results show that the Basis + DIST feature set performs better in the experiments carried out inside the gymnasium, while the set proposed here is better suited for the outdoors environment. The simple basis set without the distance features has inferior performance in all experiments in comparison with the other two features sets. In the moving scenarios of the gymnasium experiment, the Basis + DIST feature set provides a 1.5% increase in recall and precision when compared to the proposed one. On the other hand, outdoors, our proposed set provides an improvement of more than 3.5% in recall and more than 2% in precision over the Basis + DIST set performance. These findings are also evident in the DET plot in Figure 7a, where it is shown that the proposed feature set provides better recall rates over the complete operating range in the outdoors experiment. At the same time, the Basis + DIST set offers an increase over the basis set of more than 10% in recall and 3.5% in precision for the gym experiment, but only a smaller increase in recall of 3.4% outdoors, affirming that distant-dependent features are less important in outdoor scenarios. Detailed metrics are shown in Table 2.

On the second experiment, the training and test sets correspond to different scenarios. In this case, the importance of using distance-independent features becomes more apparent. As observed in Figure 7b, the proposed feature set generalizes and performs better than the Basis + DIST set for the whole operating range. Specifically, when the outdoor data were used as the training set and the gymnasium data as the test set, the gain in recall is more than 10% for false positive rates less than 10^−2^. From the same figure, it can be deduced that the normal basis set performs very similarly to the Basis + DIST set and even better in the critical operational range between 10^−3^ and 10^−2^. This further solidifies the hypotheses that the inclusion of distance-dependent features may harm the robustness of the method.

Another interesting conclusion that can be drawn from this graph is that, when data from outdoors are used for training and data from the gymnasium are used for testing, the classifiers perform significantly better than the opposite case. This was somewhat expected, since outdoor environments are generally more diverse or *difficult*. In the gymnasium experiments, the information is more canonical and cannot embrace the additional perplexity.

Generally, the results can be explained in the sense that the indoor readings are significantly confined from the surrounding structures. In a setting like this, distance-based features can be highly discriminative. On the contrary, in large outdoor environments, the same restrictions do not usually occur, and the need for a more generalized feature set becomes apparent. Additionally, in such environments, the information available is more noisy, due to sunlight, platform vibration and other uncontrolled factors; thus, the clusters are represented by a varying number of points. The proposed feature vector is able to encompass these variations, and the classifier learns to discriminate the data better. Most importantly, using our method results in a more robust training of the classifier, helping it to perform better in diverse scenarios, even if it was trained with a different dataset. This aspect is crucial for applications in autonomous large infrastructure surveillance.

Finally, it should be noted that although the feature vector in the proposed set is much larger, the execution speed is the same or even better. This happens because in the real time applications, only the feature subset chosen by the trained AdaBoost classifier needs to be extracted instead of the whole feature set.

### 8.2. Fusion Results

For the fusion experiments, only the two moving scenarios were taken into account. Comparisons are considered in two domains: first, between the performance of individual sensors and the three fusion techniques—maximum, mean, Bayes—presented in Section 6.2; second, between the fixed and proposed adaptive projection methods. Data sets are shown in Table 4, and their difference is explained in Section 7.3.

Some common observations can be made from the results of both experiments in Tables 7 and 8. First of all, it should be pointed out that since the datasets for the adaptive and fixed-size projections are of different sizes, direct comparison of the results cannot be entirely conclusive. Nevertheless, the two datasets are highly correlated, because they are obtained by the same raw data. Additionally, the dataset of the adaptive method should be considered of higher complexity, not only because of its larger size, but because it includes samples lying at further distances from the sensors. This increase in maximum distance, from 12.5 *m* to 15.63 *m*, generally results in more noisy data, thus rendering the classification task more difficult.

Equivalently to the laser-only detection, the gymnasium scenario turned out to be *easier* than outdoors. What also becomes apparent is that fusion methods have better detection rates than the individual detectors in every case. More specifically, over the laser-only approach, all metrics are improved with mean and Bayesian fusion. Maximum fusion is primarily shown for comparison. However, it can be considered a reasonable choice when high recall is of great importance or when the specificity and precision of the individual classifiers is high, as observed in the gymnasium experiments.

Another finding is that the laser recall is decreased when the adaptive projection is used. This can be mainly attributed to the increased detection range, which allows more *hard* clusters to be evaluated. Less information is available for the clusters that are far away, so it is more difficult to classify them. Despite that fact, the vision module performs even better in this case, manifesting an important advantage of the adaptive approach. In both experiments, the improvement over the fixed-size method is more than 4%.

**Figure 8.**
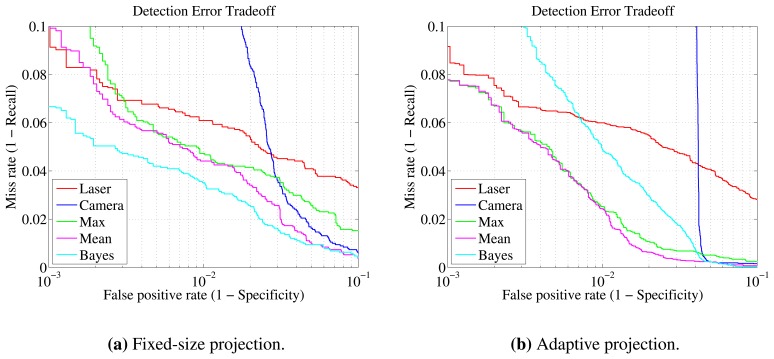
Gymnasium experiment detection error tradeoff curves The curves are plotted for each sensor individually and, also, for the three fusion techniques. Fixed-size **(a)** and adaptive **(b)** projection techniques are compared.

In the gymnasium experiment, the gains by fusion are moderate. The individual classifiers are already very accurate. Nevertheless, using the fixed projection, an increase of almost 2% in recall can be seen, and with the proposed method, the increase is slightly less than 4.5%. Precision and specificity improvement, over the laser results, in both cases, is more than 2.5%. It needs to be clarified that the results of mean and Bayesian fusion appear to be the same because the same threshold value has been used (0.5). Their quantitative and qualitative differences are depicted in detail in the DET curves of Figure 8. From these plots, it can be deduced that Bayesian fusion is better when the fixed projection size is used with a miss rate of 3.5% at a 10^−2^ false positive rate. With the adaptive projection, the mean rule provides the best tradeoff, with a 2.4% miss rate at the same false positive rate. Note that direct comparison with the individual sensor curves is not applicable, since the operating point can be adjusted independently for each one.

**Figure 9.**
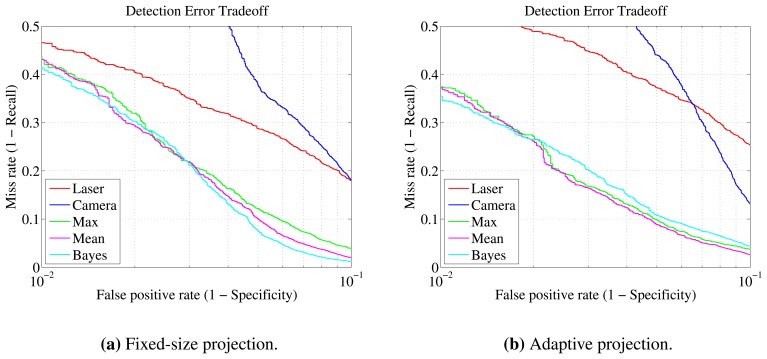
Outdoors experiment detection error tradeoff curves The curves are plotted for each sensor individually and, also, for the three fusion techniques. Fixed-size **(a)** and adaptive **(b)** projection techniques are compared.

The fusion in the outdoors experiment, in general, and the adaptive projection method, in particular, demonstrate a considerable increase in metrics. As far as individual classifier performance is concerned, when the adaptive projection is used, the vision module performance increases by more than 4.3%, while the laser module recall drops by more than 5%. This is because the laser module is heavily affected by the inclusion of further, more difficult to classify, clusters. Fusion with the fixed-width projection improves recall, precision and specificity by more than 10.4%, 13.4% and 3.4%, respectively. In the adaptive projection, recall is boosted by more than 18.5%, precision by 13.5% and specificity by 3.2%. The most prominent improvement in recall is when the maximum fusion ruled is used. In this case, the increase is almost 22% for the fixed-window projection and greater than 28% for adaptive projection. Detailed results can be found in Table 8. Similarly to the gymnasium experiment, the Bayesian rule gives better tradeoff curves when the fixed-size projection is used and the mean rule in the adaptive method, as can be observed in Figure 9. Overall, the advantage of the adaptive projection is twofold: significantly improving the fusion results, while providing further detections and a larger operating range.

**Figure 10.**
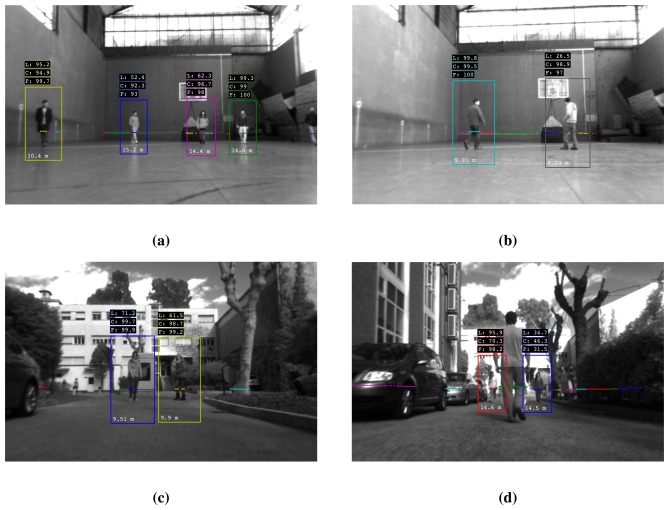
Detector images from the gymnasium **(a)**, **(b)** and outdoor experiments **(c)**, **(d)**. The boxes above each cluster give the probability of the laser and image detection modules and the mean fused probability. Cluster distance is shown at the bottom of the enclosing rectangle. **(a)** Typical scenario with multiple people, movement in various directions and occlusions. **(b)** The laser module does not detect the right person correctly. Due to correct camera classification, the person is eventually detected after fusion. **(c)** Outdoors, the laser detector performance is considerably lower. **(d)** The right person (blue rectangle) is misclassified from both sensor modules. The person in the middle is too close to the camera; in this case, the cluster is evaluated by the laser classifier only. Persons hidden by the bench on the right are not detected by the laser.

The visualization of the detection algorithm in Figure 10 gives a qualitative view of the proposed methodology. They include various people crossing each other and moving along the surroundings, in both experimental scenes.

Since the system is designed to be used in real-time surveillance, computational efficiency is also of high importance. The results presented here were produced by offline processing of data using a prototype of the method. At the same time, a speed optimized version was developed. Although not yet fully deployed, preliminary tests show an execution rate of more than 8 Hz, depending mainly on the number of clusters and, to a lesser extent, on their distance from the camera.

Certainly, our approach shows some shortcomings. First, the detection range is restricted by the vision sensor resolution. Fortunately, this can be resolved by utilizing another sensor. The cost in speed will not be significantly affected, due to the downscaling step at the beginning of Algorithm 2. Moreover, uneven terrain levels and rapid accelerations of the mobile platform can be addressed by the adaptive projection method only to a certain extent.

## 9. Conclusions

This work has demonstrated that it is feasible to accurately detect human presence from a robotic mobile platform using information from both laser and vision sensors. It is observed that meaningful fusion significantly improves the performance over single sensor detection. Furthermore, an adaptive method to address the issue of misalignment of image projection is introduced. This method was shown to outperform fixed-size projection methods, while providing longer and further detection range. For the laser detection domain, a scene independent feature set based on state-of-the-art characteristics has been created. By utilizing normalization techniques, the classifier learns feature variations. It is also observed that the proposed feature set performs and generalizes better than previous approaches. Finally, a classifier probability calibration step was introduced in order to facilitate the information fusion from the two modules. The effectiveness of our method was verified through extensive tests on real world data, which were collected from diverse experimental scenarios.

Future work will be focused on integrating the input from the fusion detector within a tracking framework for developing a human following system. Vision data could also be exploited to distinguish among different people being tracked. Moreover, extending the method for use with a distributed multi-robot system is an ongoing line of work in our research group.

## Figures and Tables

**Table 1.** List of the laser feature basis.

1. Number of points	8. Boundary length
2. Standard deviation from centroid	9. Boundary regularity
3. Mean average deviation from median	10. Mean curvature
4. Width	11. Mean angular difference
5. Linearity	12. Kurtosis
6. Circularity	13. Aspect ratio
7. Radius	

**Table 2.** Results comparison of laser feature sets Three feature sets are compared. The feature basis is described in Table 1. DIST denotes the addition of distance features to the feature basis. The proposed feature set is described in Section 4.2. The laser data sets from Table 3 were used.

	**Static**			**Moving**		

	**Basis**	**Basis + DIST**	**Proposed**	**Basis**	**Basis + DIST**	**Proposed**
**Precision**	95.25%	98.89%	97.86%	86.67%	91.26%	89.75%
**Recall**	88.60%	98.67%	96.96%	84.66%	90.14%	88.57%
**Specificity**	99.75%	99.94%	99.88%	98.27%	98.86%	98.66%

(a) Gymnasium experiments.						

	**Moving**		

	**Basis**	**Basis + DIST**	**Proposed**
**Precision**	76.84%	76.83%	78.97%
**Recall**	59.93%	63.36%	66.11%
**Specificity**	97.97%	97.83%	98.00%

(**b**) Outdoor experiments.			

**Table 3.** Overview of the acquired data set. Three scenarios in two different places were collected in total.

	**Gymnasium**		**Outdoors**	**Total**

	**Static**	**Moving**	**Moving**	
**Scans**	10,350	10,800	4,000	25,150
**Total segments**	191,418	191,426	102,576	485,420
**Human segments**	10,179	22,412	10,449	43,040
**Seconds**	258.75	270	100	628.75
**Persons appearing**	6	12	10	28

**Table 4.** Data sets used for fusion detection. Different ROI projection sizes lead to different data sets.

	**Fixed-size ROI**	**Adaptive ROI**
**Total segments**	12,686	18,504
**Human segments**	10,780	12,531

(**a**) Gymnasium experiment (moving scenario).		

	**Fixed-size ROI**	**Adaptive ROI**
**Total segments**	27,261	30,543
**Human segments**	6480	6611

(**b**) Outdoors experiment.		

**Table 5.** Data sets used for support vector machine (SVM) classifier training.

	**Preliminary Training**	**Final Training**	**Testing**
**Positive** (Human) [INRIA,MIT]	3,340	3,340	1,126
**Negative** (Non-human) [INRIA]	12,800	47,229	4,530

**Table 6.** Confusion matrix of the final HoG-SVM classifier on the INRIA test set. The percentages in parenthesis are computed horizontally and correspond to TPR, FNR, FPR and TNR, respectively.

		**Predicted Label**	

		**Human**	**Non-human**
**Actual**	**Human**	1,071 (95.12%)	55 (4.88%)

	**Non human**	40 (0.88%)	4,490 (99.12%)

**Table 7.** Gymnasium experiment results Comparison of the fixed (**a**) and the proposed adaptive (**b**) projection methods. The performance of using each sensor individually is also compared with three different information fusion techniques. The data sets used for these results are shown in Table 4a.

	**Individual Sensors**		**Information Fusion**		

	**Laser**	**Camera**	**Maximum**	**Mean**	**Bayes**
**Precision**	99.29%	99.85%	99.26%	99.82%	99.82%
**Recall**	92.61%	91.33%	98.15%	94.58%	94.58%
**Specificity**	96.28%	99.21%	95.86%	99.01%	99.01%

(**a**) Fixed-size projection.					

	**Individual Sensors**		**Information Fusion**		

	**Laser**	**Camera**	**Maximum**	**Mean**	**Bayes**
**Precision**	98.44%	99.54%	98.44%	99.88%	99.88%
**Recall**	90.72%	95.68%	99.27%	95.20%	95.20%
**Specificity**	97.12%	99.05%	96.65%	99.76%	99.76%

(**b**) Adaptive projection.					

**Table 8.** Outdoor experiment results Comparison of the fixed (**a**) and the proposed adaptive (**b**) projection methods. The performance of using each sensor individually is also compared with three different information fusion techniques. The data sets used for these results are shown in Table 4b.

	**Individual Sensors**		**Information Fusion**		

	**Laser**	**Camera**	**Maximum**	**Mean**	**Bayes**
**Precision**	86.22%	87.17%	85.51%	99.63%	99.63%
**Recall**	69.43%	83.15%	91.17%	79.83%	79.83%
**Specificity**	96.54%	96.18%	95.18%	99.99%	99.99%

(**a**) Fixed-size projection.					

	**Individual Sensors**		**Information Fusion**		

	**Laser**	**Camera**	**Maximum**	**Mean**	**Bayes**
**Precision**	85.02%	86.42%	81.55%	98.58%	98.58%

**Recall**	64.38%	87.52%	93.01%	82.95%	82.95%

**Specificity**	96.87%	96.20%	94.19%	99.67%	99.67%

(**b**) Adaptive projection.					
